# Real-Time Intelligent Coaching Versus Human Expert Instruction in Surgical Skills Training: A Systematic Review and Meta-Analysis

**DOI:** 10.7759/cureus.115874

**Published:** 2026-09-06

**Authors:** Syed Taqi Askari Shah, Yashar Mashayekhi, Nidhi Reji, Fatima Nawaz, Abdul Rehman, Mandeep Kumar, Ayodele Odedara, Ali Saif Khalfan Alkaabi, Seungyoung Kang, Basel Bashar Abdulrahem Mousa, Syed Muhammad Ali, Maheen Qazi

**Affiliations:** 1 Trauma and Orthopedics, Queen's Medical Centre, Nottingham University Hospitals NHS Trust, Nottingham, GBR; 2 Trauma and Orthopaedics, University Hospitals of Leicester NHS Trust, Leicester, GBR; 3 General Surgery, Rotherham General Hospital, The Rotherham NHS Foundation Trust, Rotherham, GBR; 4 Medicine, Shifa College of Medicine, Shifa Tameer-E-Millat University, Islamabad, PAK; 5 General Dentistry, Shifa College Of Dentistry, Rawalpindi, PAK; 6 Medicine, Jinnah Medical and Dental College, Karachi, PAK; 7 Trauma and Orthopaedics, Royal Shrewsbury Hospital, Shrewsbury, GBR; 8 Internal Medicine, Mohammed Bin Rashid University of Medicine and Health Sciences, Dubai, ARE; 9 Medicine, Thumbay University Hospital, Ajman, ARE; 10 Internal Medicine, Kharkiv National Medical University, Kharkiv, UKR; 11 Surgery, Weill Cornell Medicine - Qatar, Doha, QAT; 12 Acute Care Surgery, Hamad General Hospital, Doha, QAT; 13 Pediatric Surgery, Royal Hospital for Children and Young People, Edinburgh, GBR

**Keywords:** artificial intelligence, cognitive load, medical students, meta-analysis, randomized controlled trials, surgical education

## Abstract

Real-time intelligent coaching systems are technology-based surgical training tools that provide automated, real-time, and objective feedback on learners’ performance during simulated surgical tasks. Unlike human expert instruction, which relies on instructor-delivered guidance and feedback, these systems use automated performance monitoring to provide immediate feedback on aspects such as accuracy, motion economy, and errors. This systematic review and meta-analysis investigated the effectiveness of real-time intelligent coaching systems compared with human expert training in surgical skill instruction, with technical surgical performance as the primary outcome and cognitive load and emotional outcomes as secondary outcomes. We followed the PRISMA guidelines and conducted a comprehensive search of PubMed, the Cochrane Library, ClinicalTrials.gov, and ScienceDirect through March 2026. We considered randomized controlled trials including medical students with no prior surgical experience. We measured outcomes using the Intelligent Continuous Expertise Monitoring System (ICEMS) and the Objective Structured Assessment of Technical Skills (OSATS) scores, emotional responses, and cognitive load. We included three randomized controlled trials involving 281 participants. No statistically significant differences were observed between real-time intelligent coaching systems and human expert instruction in technical performance, as measured by ICEMS and OSATS scores; however, the ICEMS analysis showed very high heterogeneity (I² = 99%), and only two trials reported technical outcomes.

Real-time intelligent coaching systems were associated with a small reduction in overall cognitive load compared with human expert instruction, with the intrinsic cognitive load domain also favoring real-time intelligent coaching systems. No significant differences were observed in positive activating or negative activating emotions, whereas positive deactivating emotions were modestly higher with real-time intelligent coaching systems. Given the small number of studies, limited sample sizes, and substantial heterogeneity, interpret the findings cautiously. Current evidence suggests that real-time intelligent coaching systems may provide technical performance similar to human expert instruction and may offer some cognitive benefits, but further well-designed, adequately powered trials are needed to establish their effectiveness and role in surgical education.

## Introduction and background

Conventionally, human instructors have conducted surgical training through an apprenticeship-based approach in which trainees acquire skills [1]. Although this has been the backbone of training, growing concerns about limited operating room availability, patient safety, and teaching quality have prompted more formalized, systematic training [2,3]. Meanwhile, recent advances in Artificial Intelligence and digital simulation technologies have enabled the creation of real-time intelligent coaching systems that can aid in learning surgical skills [4,5]. In this review, real-time intelligent coaching systems refer to technology-based training systems that provide automated, real-time, and objective feedback on learners’ surgical performance, including measures such as accuracy, motion economy, and errors, during simulated surgical tasks.

These systems provide real-time, objective, and dependable feedback and are often integrated into virtual reality or video-based applications [5,6]. Unlike traditional instruction, which is subjective and instructor-heavy, real-time coaching platforms apply machine learning algorithms to performance outcome measures, including motion economy, accuracy, and error rates [7]. These systems have emerged as potential adjuncts to, rather than replacements for, human expert instruction in surgical training [6].

However, the performance of such systems must be evaluated not only in terms of competency but also in terms of cognition [8]. Surgical training, a process that comprises complex psychomotor and cognitive processes, applies to cognitive load theory [9]. Excessive cognitive load may interfere with learning, but properly designed instruction systems may enhance performance by reducing extraneous cognitive load [10]. Cognitive load can be considered across three dimensions: intrinsic cognitive load, which reflects the inherent complexity of a task and the expertise of the learner; extraneous cognitive load, which results from processes that do not directly contribute to learning; and germane cognitive load, which reflects learning processes that deal with intrinsic cognitive load [9]. Technical performance in surgical training can be assessed using the Intelligent Continuous Expertise Monitoring System (ICEMS) and the Objective Structured Assessment of Technical Skills (OSATS), which provide standardized measures of surgical technical performance [11,12]. Although early studies have compared intelligent coaching systems with human expert instruction, evidence regarding their effects on technical performance and cognitive load remains limited and heterogeneous [13,14]. Recent systematic reviews published after the present search period have further highlighted the growing evidence base for artificial intelligence in surgical education, while also underscoring the limited number of comparative trials and the need for further rigorous evaluation of cognitive and performance outcomes in AI-assisted surgical training [15,16].

Although primary studies have examined intelligent coaching systems in surgical training, findings remain inconsistent because of differences in study design, training methods, feedback approaches, and outcomes [7,14]. Therefore, this systematic review and meta-analysis aimed to compare real-time intelligent coaching systems with human expert instruction among medical students without previous surgical experience undergoing simulation-based surgical skills training, focusing on technical performance, cognitive load, and emotional outcomes.

Research gap

Although interest in technology-based surgical education is increasing, evidence directly comparing real-time intelligent coaching systems with human expert instruction remains limited. Existing studies have small sample sizes and vary in surgical tasks, training methods, feedback approaches, and outcome measures. Furthermore, cognitive load, an important factor in learning efficiency and retention, has received less attention than technical skill acquisition. Therefore, uncertainty remains regarding the comparative effects of real-time intelligent coaching systems and human expert instruction on technical performance, cognitive load, and emotional outcomes among medical students undergoing simulation-based surgical training.

Study objectives

Primary Objective

To determine whether real-time intelligent coaching systems differ from human expert instruction in technical surgical skill performance, assessed using ICEMS and OSATS scores, among medical students undergoing simulation-based surgical training.

Secondary Objective

To determine whether real-time intelligent coaching systems differ from human expert instruction in cognitive load and emotional outcomes during simulation-based surgical training.

## Review

Methods

This meta-analysis was conducted according to the Preferred Reporting Items for Systematic Reviews and Meta-Analyses (PRISMA) guidelines, and the protocol of this systematic review was registered on PROSPERO (Registration No: CRD420261355901) [17].

Search Strategy

We searched 4 databases: PubMed, The Cochrane Library, ClinicalTrials.gov, and ScienceDirect. We designed a search strategy for each database using the Boolean operators ‘AND’ and ‘OR’. For PubMed, we used ‘((((Artificial Intelligence Tutoring) OR (AI tutoring)) AND (Expert instruction)) OR (human instruction)) AND (surgical skills)’, for Cochrane, we used ‘(Artificial Intelligence Tutoring): ti, ab,kw OR (AI tutoring): ti, ab,kw AND (Expert instruction): ti, ab,kw OR (human instruction): ti, ab,kw AND (surgical skills): ti, ab,kw’, for Science Direct ‘(((Artificial Intelligence Tutoring) OR (AI tutoring)) AND ((Expert instruction) OR (human instruction)) AND (surgical skills))’ and same search strategy as PubMed was used for clinicaltrials.gov. We searched only for trials from inception through March 2026. We also searched the reference lists of the included articles for studies. The detailed search strategy is available in supplementary information Table 2.

Eligibility Criteria

We included studies involving medical students with no prior surgical experience from the pre-clinical years of medical school (i.e., years 1, 2, and 3) who gave consent and demonstrated understanding of the study protocols. The intervention was artificial intelligence-based feedback on surgical skills, and the comparator was feedback from expert surgeons in the field. Articles had to report at least one primary outcome, i.e., the ICEMS expertise score and OSATS, to be included. We measured cognitive load using the Cognitive Load Index (CLI), a 5-point Likert scale, with higher scores indicating greater cognitive load. We included only trials published in English.

We excluded studies that included medical students with prior surgical experience, either from workshops or from their clinical rotations (i.e., years 4 and 5). Studies with no information on student consent were also excluded. Studies were also excluded if AI and expert feedback were combined rather than analyzed separately. We excluded studies in languages other than English, as well as cohort studies, cross-sectional studies, case studies, conference abstracts, reviews, and letters. We also excluded studies that did not report on either ICEMs or OSATS.

Selection and Data Extraction

We exported all retrieved articles to EndNote for duplicate checking and manually removed duplicates. We screened the remaining articles by title and abstract. Articles deemed relevant were searched for full-text availability, and those with full text underwent full-text screening, during which eligibility criteria were applied. Studies fulfilling our criteria were included in the results. Two reviewers carried out the entire process independently, and any disputes about an article's inclusion were resolved with the help of a third reviewer.

Data were extracted into an Excel sheet and included author names, year, gender, sample size, age, intervention, comparator, and outcome details. Two reviewers carried out this process, and a third reviewer reviewed it; external help was sought for methodology and results.

Quality Assessment

We assessed the quality of the included RCTs using Cochrane's ROB2 risk-of-bias tool. We graded the RCTs as high, some concerns, or low quality. A low-risk study was one in which all 5 domains showed low risk of bias. Studies with some concerns had one domain of concern, and the remaining domains were low risk. Similarly, studies with a high risk of bias had at least 2 domains with some concerns or at least one domain with high risk of bias [18].

Data Synthesis

We used RevMan 5.4 to analyze the included studies [19]. We used mean differences and standard deviations for continuous outcomes, with 95% confidence intervals based on a random-effects inverse-variance model. I2 was used as a predictor of statistical heterogeneity, with heterogeneity exceeding 40% considered significant. Heterogeneity above 75% was considered high, 50-75% moderate, 26-50% mild, and below 25% low. Forest plots were used to present the results. A p-value below 0.05 was considered significant. Sensitivity analysis, subgroup analysis, and a funnel plot to assess publication bias could not be performed due to the limited number of studies and available data.

Results

Our database searches yielded about 1341 articles, with the majority from PubMed. We performed a duplicate search and excluded 211 articles. Of the remaining 1130 articles screened by title and abstract, we removed 1098 as irrelevant. Of the remaining 32 articles, we could not find the full text of 3, and of the remaining 29, only 3 met our eligibility criteria [20-22]. We did not find any relevant articles in the reference lists of the included articles. The whole screening process is summarized below in Figure 1.

**Figure 1:**
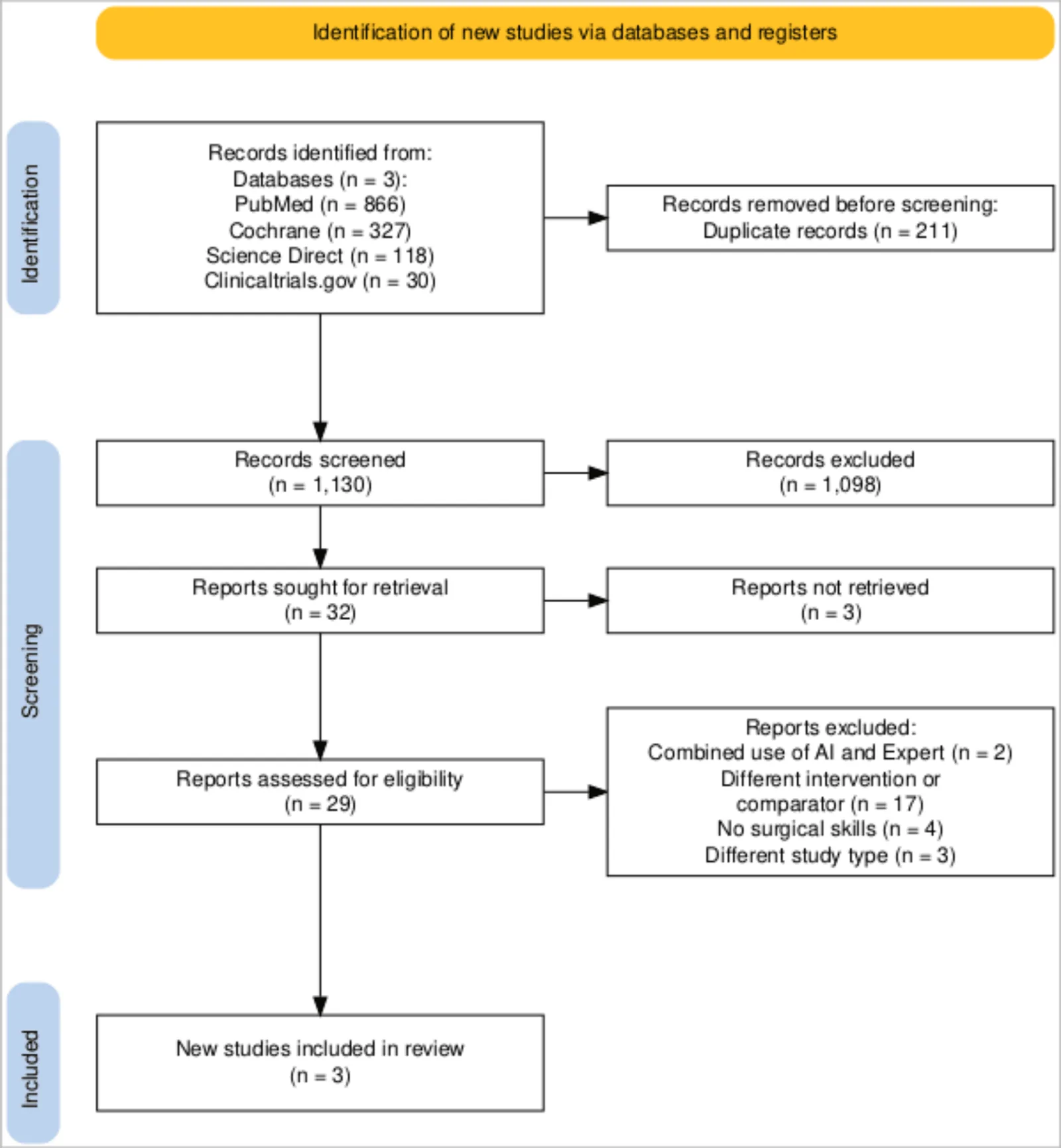
PRISMA flowchart summarizing screening and selection Flowchart illustrating the systematic process of literature identification, screening, eligibility assessment, and inclusion of studies in the meta-analysis. A total of three randomized controlled trials were included after applying eligibility criteria.

Characteristics of Studies and Quality of Studies

We included three RCTs in the analysis, and all of them were instructor-blinded. Our review included 281 participants: 94 in the AI tutor group, 94 in the expert tutor group, and 93 in the control group. The mean age in our review was 22.70 ± 2.88, with most participants female (159 females, 121 males). Two studies included students in years 1 and 2, one study included participants in year 3 only, and all students had no prior surgical experience. Most participants were right-handed (255), with 25 left-handed and one ambidextrous. Table 1 summarizes the characteristics of the included studies.

**Table 1:** Characteristics of the included studies

Study ID	Design	Sample Size	Age (Medical Students)	Male: Female: Prefer not to say (Medical Students)	Year of Study	Dominant Hand (Medical Students)	Type of AI	Primary Outcomes	Key Findings
Sun Y et al. [20]	Single-Blinded RCT	AI tutor = 41 Human Tutor = 41 Self-Learning = 42	23.2 ± 2.0	52: 72: 0	Year 1 = 0 Year 2 = 0 Year 3 = 124	Right = 112 Left = 12 Ambidextrous = 0	MetaGP-SurGED	OSATS, FLS score, emotions and cognitive load	No significant difference between AI tutor and Human Tutor
Fazlollahi MA et al. [21]	Single-Blinded RCT	AI tutor = 23 Human Tutor = 24 Self-Learning = 23	21.8 ± 2.3	29: 41: 0	Year 1 = 51 Year 2 = 19 Year 3 = 0	Right = 66 Left = 4 Ambidextrous = 0	Virtual operative assistant AI tutoring	ICEMS, OSATS, emotions and cognitive load	Performance scores were better with AI tutor than with human tutor
Giglio B et al. [22]	Single-Blinded RCT	AI tutor = 30 Human Tutor = 29 AI + Human Tutor = 28	22.7 ± 4.0	40: 46: 1	Year 1 = 67 Year 2 = 20 Year 3 = 0	Right = 77 Left = 9 Ambidextrous = 1	Real-time verbal feedback directly from the ICEMS	ICEMS, self-reported cognitive responses, Emotions and cognitive load	Higher performance scores with human expert than AI

All of the included studies were low risk as assessed by ROB2, with low risk across all domains, as summarized in Figure 2. The outcome-level assessment is provided in the supplemental information (Table 3).

**Figure 2:**
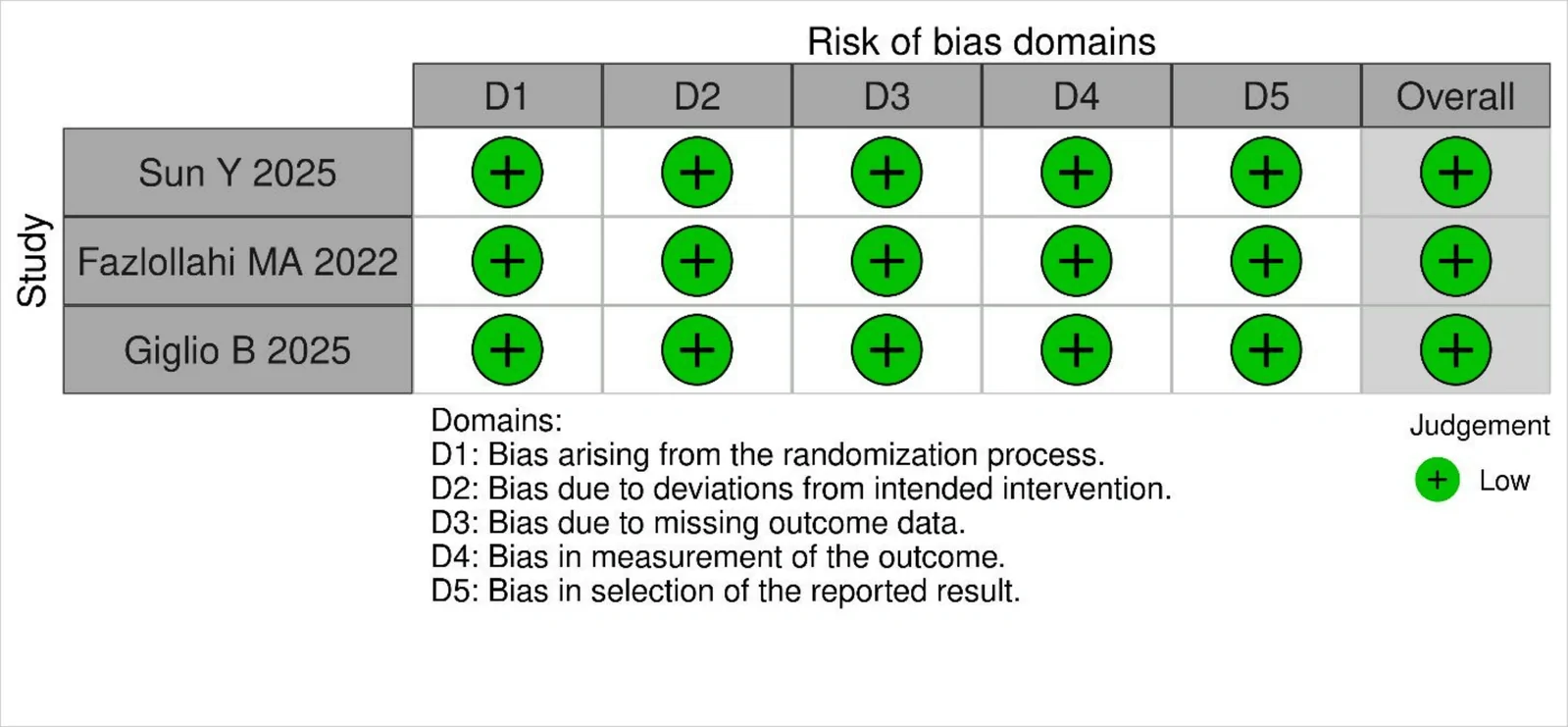
Summary of risk of bias Graphical summary of the risk of bias across included randomized controlled trials assessed using the Cochrane ROB2 tool. All included studies demonstrated an overall low risk of bias across evaluated domains. References: Sun Y et al. [20]; Fazlollahi MA et al.  [21]; Giglio B et al. [22].

ICEMS and OSATS

Two RCTs reported ICEMS scores comparing AI feedback with expert tutor feedback. In the study by Fazlollahi et al. (2022) [21], the experimental group had a mean expertise score of 0.14 compared with −0.62 in the control. In Giglio et al. (2025) [22], the corresponding means were −0.32 and −0.20, respectively. Across the two studies, the direction and magnitude of effects were inconsistent. Thus, although one study showed a numerically higher ICEMS expertise score with AI feedback, the overall evidence did not demonstrate a clear improvement.

Two RCTs also reported OSATS scores comparing AI feedback with expert tutor instruction. In Fazlollahi et al. (2022) [21], the mean OSATS score was 4.63 in the AI group versus 4.40 in the comparator group, while Sun Y et al. (2025) [20] reported mean scores of 3.90 and 4.20, respectively. Overall, the available studies did not show a clear advantage of AI feedback over expert tutor instruction in OSATS-assessed technical skill.

The GRADE assessment for our primary outcomes is available in the supplementary information (Table 4).

Effect on Emotions

All trials reported on the emotional aspect, including positive activating, negative activating, and positive deactivating. Our analysis showed that AI group increased positive activating scores but the results were not significant (MD 0.15; CI -0.00, 0.31, p = 0.05), no significant difference was observed between both groups in terms of reducing negative activating emotions (MD -0.05; CI -0.19, 0.09, p = 0.47) however, a significant increase in positive deactivating emotions was observed with AI group (MD 0.16; CI 0.03, 0.28, p = 0.02). No statistical heterogeneity was observed across all results. Figure 3 shows a forest plot of these results.

**Figure 3:**
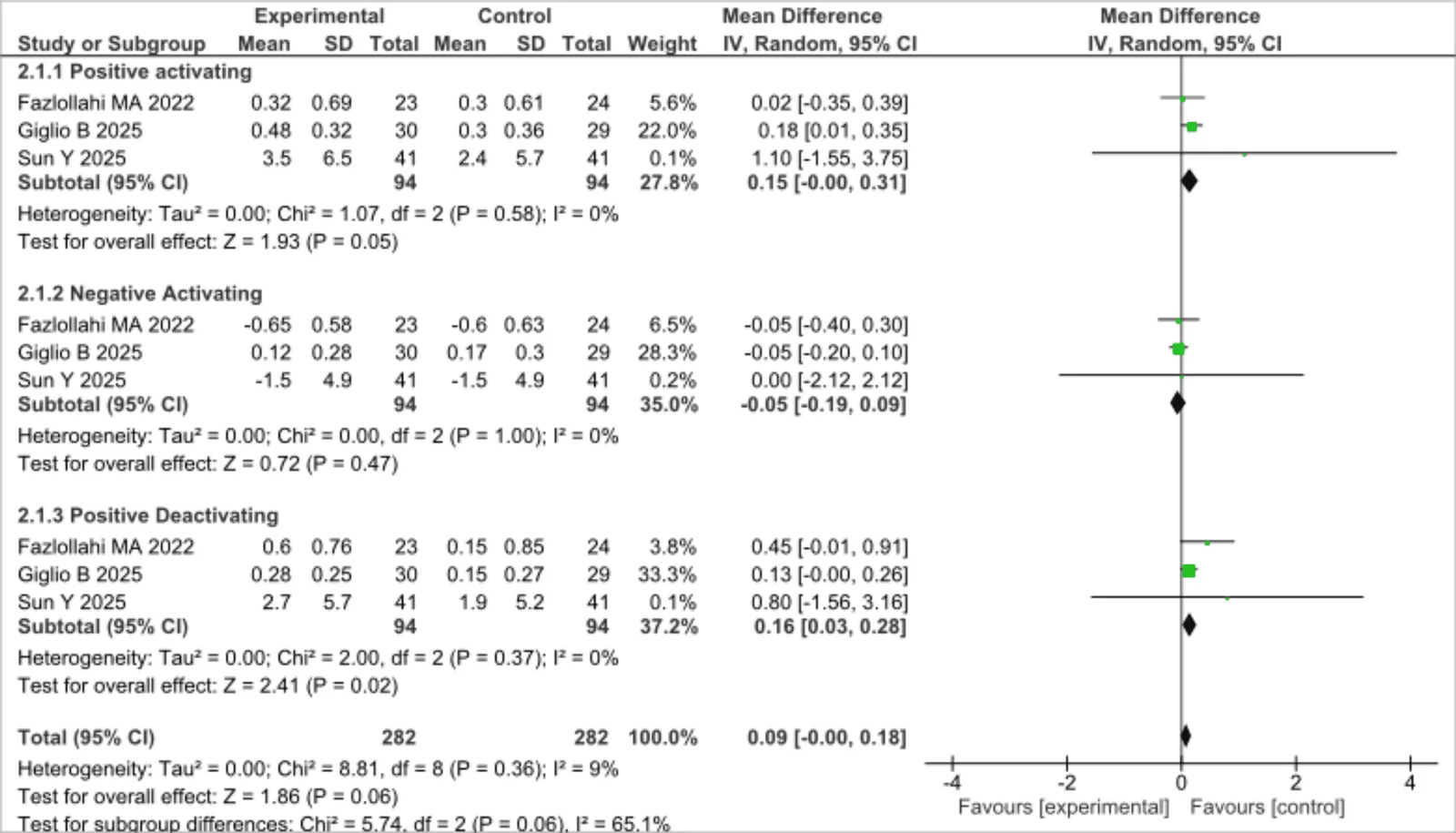
Forest plot showing differences in emotional aspects between the AI and the expert tutor group References: Sun Y et al. [20]; Fazlollahi MA et al.  [21]; Giglio B et al. [22].

Cognitive Load

All RCTs reported on cognitive load, and a significant difference was observed between the groups, with the expert tutor group experiencing greater cognitive load (MD = -0.17; CI = -0.32, -0.01, p = 0.03). However, no significant difference was observed in extrinsic (MD = -0.10; CI = -0.52, 0.31, p = 0.62) and germane factors (MD = -0.21; CI = -0.44, 0.01, p = 0.07) of cognitive load. For the intrinsic factor, a significant improvement was observed in the AI group, with no statistical heterogeneity (MD = -0.27; CI = -0.52, -0.03, p = 0.03). Figure 4 shows a forest plot of these results.

**Figure 4:**
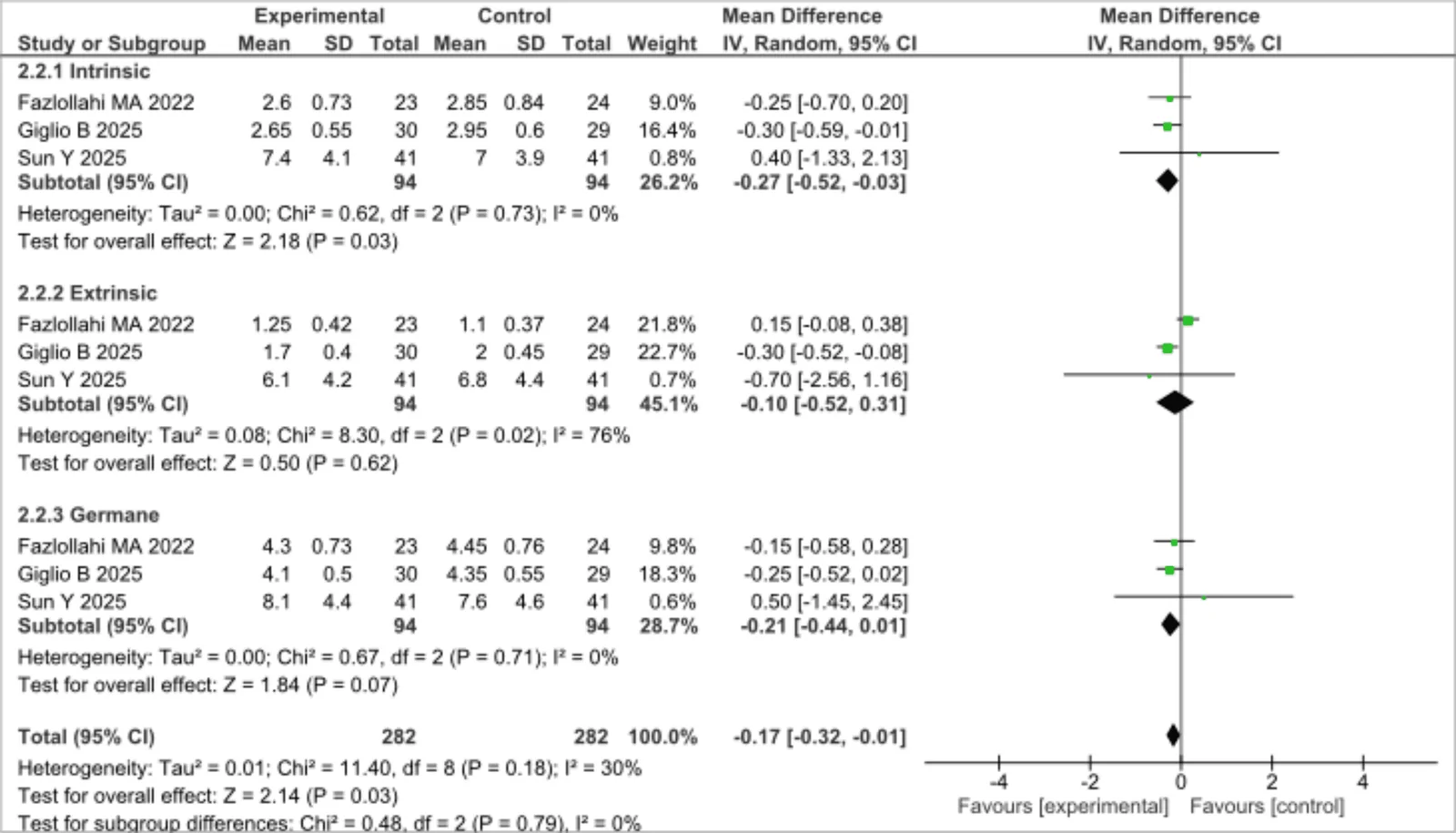
Forest plot showing differences in cognitive load between the AI and expert tutor group References: Sun Y et al. [20]; Fazlollahi MA et al.  [21]; Giglio B et al. [22].

The variation in study weights for emotion and cognitive outcomes reflects differences in the precision of the individual effect estimates, which are determined by sample size and variability (SD), rather than sample size alone.

Discussion

The present meta-analysis found no statistically significant difference between real-time intelligent coaching systems and human expert instruction in technical surgical performance, while suggesting a small reduction in overall cognitive load with intelligent coaching. However, these findings should be interpreted cautiously because only three randomized controlled trials met the eligibility criteria, technical performance outcomes were available from only two studies, and the ICEMS analysis demonstrated very high heterogeneity (I² = 99%). These limitations indicate substantial uncertainty around the pooled estimates and preclude concluding that the two instructional approaches are equivalent in effectiveness.

Regarding technical performance, several randomized trials found no statistically significant difference between AI-based feedback and expert instruction, but some point estimates favored real-time intelligent coaching systems; however, these results were inconsistent across studies. Differences in training design, feedback delivery, and system architecture may explain why statistical significance is not uniform across analyses and why heterogeneity is high [5,21]. Heterogeneity is extremely high and could be explained by differences in the design of real-time intelligent coaching systems, surgical activity, feedback, and training time across studies. Similarly, the OSATS scores showed no statistically significant difference between real-time intelligent coaching systems and human expert instruction. However, this finding should not be interpreted as evidence of equivalent effectiveness. These findings are consistent with other randomized trials and systematic reviews that have reported similar performance with AI-aided feedback and instructor-led learning in simulation-based surgical training [4,6].

The very high heterogeneity observed for ICEMS (I² = 99%) substantially limits the clinical interpretability of the pooled estimate (MD = 0.32), and this result should therefore be interpreted with considerable caution. Similarly, although an overall pooled estimate was generated for emotional outcomes, positive activating, negative activating, and positive deactivating emotions represent conceptually distinct constructs. The near-significant subgroup difference (p = 0.06; I² = 65.1%) suggests that these outcomes may not share a sufficiently homogeneous effect and should therefore be interpreted primarily as separate emotional domains rather than as a single omnibus effect. In contrast, the cognitive-load domains showed no evidence of a subgroup difference (p = 0.79), making their pooled interpretation comparatively more defensible.”

In the affective domain, AI feedback correlated with a nonsignificant positive effect on activating emotion. This suggests a small but potentially meaningful change in learners' engagement. The findings align with existing literature indicating that AI-based feedback can influence emotional activation with a small positive effect. Nevertheless, it does not have a homogeneous and context-independent effect [23,24]. Additionally, no statistically significant difference was observed in negative activating emotions between real-time intelligent coaching systems and human expert instruction [21,25]. Our study also showed that positive deactivating emotions (calmness and contentment) improved significantly under AI feedback. This finding is also broadly consistent with evidence from general educational settings suggesting that AI-based interventions may influence learners’ emotional states. However, the relevance of these findings to simulated surgical training remains uncertain [26,27]. This finding may relate to the structured and standardized nature of automated feedback; however, the included studies did not directly evaluate this explanation. Nevertheless, these results should be viewed with caution, as the emotional results were secondary and the effect sizes were minimal.

In terms of cognitive load, the expert-tutor group showed a marginally higher overall cognitive load than the real-time intelligent coaching system group. This finding may be broadly consistent with cognitive load theory and evidence from general educational settings, although direct evidence from surgical training remains limited [28]. In contrast, AI-based systems can alleviate extraneous cognitive load by providing structured, task-specific, real-time feedback. No statistically significant differences were observed between real-time intelligent coaching systems and human expert instruction in extraneous and germane cognitive load. Although the AI group showed a small reduction in overall cognitive load, the intrinsic cognitive load analysis also favored the AI group (MD = −0.27; 95% CI, −0.52 to −0.03; p = 0.03), while no significant differences were observed in extraneous or germane cognitive load. This implies that the mode of instruction may affect different aspects of cognitive load. This interpretation is broadly consistent with findings from general educational settings suggesting that well-designed AI-based instructional systems may support learning without substantially increasing learners' cognitive load; however, the applicability of these findings to surgical training remains uncertain [29]. In addition, real-time intelligent coaching systems were associated with a decrease in intrinsic cognitive load compared with human expert instruction. This finding may suggest that real-time intelligent coaching can reduce the cognitive demands of task execution; however, the limited number of studies prevents broader conclusions about the role of AI in surgical training. This interpretation is broadly consistent with findings from general educational settings suggesting that effective interaction with AI systems may facilitate task processing and learning; however, the applicability of these findings to surgical training remains uncertain [30].

However, the findings should not be interpreted as supporting replacement of human expertise. Human instructors may provide contextual feedback, encouragement, and adaptive guidance that were not evaluated in the present meta-analysis. The findings therefore support further investigation of how real-time intelligent coaching systems might complement, rather than replace, human expert instruction.

Differences between the included studies may have been related to variation in the simulated surgical tasks, simulator characteristics (e.g., virtual reality or video-based systems), feedback methods, training duration, and outcome measures. These differences highlight the variability of current intelligent coaching technologies and the need for standardized assessment approaches.

Overall, the current research contributes to the growing literature on integrating artificial intelligence into surgical training. It highlights the potential of real-time intelligent coaching systems to support technical skill development and cognitive efficiency during simulated surgical training. However, the findings are based on only three randomized controlled trials involving medical students without previous surgical experience, which limits the statistical power and generalizability of the pooled estimates.

Limitations

This review has limitations that should be considered when interpreting the results. To begin with, the included studies were highly heterogeneous in study design, sample size, surgical procedure, and outcome measures. This variability may limit the extrapolation of the aggregate result. The high heterogeneity of key outcomes also reduces confidence in the combined estimates and limits interpretation. Second, a significant percentage of the articles included in the analysis were small in scale and may have limited statistical power and increased bias. Moreover, inconsistent definitions and measures of cognitive load, which may rely on subjective assessment tools, can affect the consistency of findings.

Third, most studies have been conducted in a simulated setting rather than a clinical environment. Training through simulation does not always reflect the stress and complexity of actual surgical practice, which limits external validity. Fourth, publication bias cannot be eliminated, as results indicating that intelligent coaching systems have a positive effect may be more likely to be published. Finally, intelligence systems are evolving so fast that some of the studies mentioned may already be out of date, thus underestimating what machines are capable of today.

Future directions

Future research should aim for large, multicenter randomized controlled trials to produce more powerful, generalizable evidence. Cognitive load and outcome measures in general should be standardized so they can be meaningfully compared across studies. The long-term effects of intelligent coaching systems on patient outcomes, whether skills transfer to real surgical settings, and skill retention should also be evaluated. The current literature measures short-term performance, creating a research gap in long-term learning behavior.

Further development of hybrid training models that combine intelligent systems and human expert training should be explored. Such methods could combine the advantages of both modalities and offer highly personalized, adaptive, and contextual training experiences.

In addition, further studies should examine the cost-effectiveness, availability, and scalability of such technologies, particularly in low-resource contexts where expert trainers may be scarce. Finally, advances in artificial intelligence, such as adaptive learning algorithms and real-time decision support, must be considered to make intelligent coaching systems more complex and clinically applicable.

## Conclusions

This systematic review and meta-analysis suggests that real-time intelligent coaching systems may achieve comparable short-term technical performance to human expert instruction during simulation-based surgical training; however, the certainty of evidence is low to very low because of the small number of trials, limited sample sizes, substantial heterogeneity for some outcomes, and imprecision of estimates. Real-time intelligent coaching systems may also be associated with a small reduction in overall cognitive load, including a significant reduction in intrinsic cognitive load, although effects varied across cognitive-load domains. The available evidence is limited to medical students without previous surgical experience performing simulated surgical tasks. It does not establish effects on operating-room performance, long-term skill retention, or patient outcomes. Therefore, real-time intelligent coaching systems should currently be considered potential adjuncts to, rather than replacements for, human expert instruction. Hybrid AI-human training models warrant further investigation in adequately powered randomised trials.

## Appendices

**Table 2:** Supplementary information.

Word	Variation
Artificial intelligence	"artificial intelligence"[MeSH Terms] OR ("artificial"[All Fields] AND "intelligence"[All Fields]) OR "artificial intelligence"[All Fields]
Tutoring	"tutor"[All Fields] OR "tutor's"[All Fields] OR "tutored"[All Fields] OR "tutoring"[All Fields] OR "tutors"[All Fields]
Expert	"expert"[All Fields] OR "expert's"[All Fields] OR "expertize"[All Fields] OR "experts"[All Fields]
Instruction	"instruct"[All Fields] OR "instructed"[All Fields] OR "instructing"[All Fields] OR "instructional"[All Fields] OR "instructions"[All Fields] OR "instructive"[All Fields] OR "instructively"[All Fields] OR "instructiveness"[All Fields] OR "instructs"[All Fields] OR "teaching"[MeSH Terms] OR "teaching"[All Fields] OR "instruction"[All Fields]
Human	human's"[All Fields] OR "humans"[MeSH Terms] OR "humans"[All Fields] OR "human"[All Fields]
Surgical	"surgical procedures, operative"[MeSH Terms] OR ("surgical"[All Fields] AND "procedures"[All Fields] AND "operative"[All Fields]) OR "operative surgical procedures"[All Fields] OR "surgical"[All Fields] OR "surgically"[All Fields] OR "surgicals"[All Fields]
Skill	"skill"[All Fields] OR "skilled"[All Fields] OR "skillful"[All Fields] OR "skillfulness"[All Fields] OR "skills"[All Fields]

**Table 3:** Supplementary information. References: Sun Y et al. [20]; Fazlollahi MA et al.  [21]; Giglio B et al. [22].

Study	Outcome	D1	D2	D3	D4	D5	Overall
Fazlollahi et al., 2022	ICEMS	Low	Low	Low	Low	Low	Low
Fazlollahi et al., 2022	OSATS	Low	Low	Low	Low	Low	Low
Giglio et al., 2025	ICEMS	Low	Low	Some concerns	Low	Low	Some concerns
Giglio et al., 2025	OSATS	N/A	N/A	N/A	N/A	N/A	N/A
Sun & Liu, 2025	ICEMS	N/A	N/A	N/A	N/A	N/A	N/A
Sun & Liu, 2025	OSATS	Low	Low	Low	Low	Some concerns	Some concerns

**Table 4:** Supplementary information.

Outcome	No. of studies	Study design	Risk of bias	Inconsistency	Indirectness	Imprecision	Other considerations	No. of patients [intervention] [comparison]	Effect MD (95% CI)	Certainty	Importance
ICEMS Expertise score	2	randomised trials	serious	very serious	not serious	serious	none	117 / 118	MD 0.32 (−0.54 to 1.18)	⊕⊕◯◯ Low	CRITICAL
OSATS	2	randomised trials	serious	not serious	not serious	serious	none	64 / 64	MD 0.08 (−0.54 to 0.70)	⊕⊕◯◯ Low	CRITICAL
